# Developing a Performance Measurement Framework and Indicators for Community Health Service Facilities in Urban China

**DOI:** 10.1186/1471-2296-11-91

**Published:** 2010-11-18

**Authors:** Sabrina T Wong, Delu Yin, Onil Bhattacharyya, Bin Wang, Liqun Liu, Bowen Chen

**Affiliations:** 1 University of British Columbia, School of Nursing and Centre for Health Services Policy Research, 6190 Agronomy Road, #302, Vancouver, British Columbia, V6T-1Z3, Canada; 2 Capital Institute of Pediatrics and Community Health Association of China. 2 YaBao Road, 328#, ChaoYang District, Beijing, 100020, China; 3 University of Toronto, Department of Family and Community Medicine and Li Ka Shing Knowledge Institute of St. Michael's Hospital, 80 Bond Street, First Floor, Toronto, Ontario, M5B 1X2, Canada; 4 Community Health Division, the Basic Health and Maternal and Child Care of Health Department, Ministry of Health of The People's Republic of China, XiZhimenWai Road 1, XiCheng District, Beijing, 100044, China

## Abstract

**Background:**

China has had no effective and systematic information system to provide guidance for strengthening PHC (Primary Health Care) or account to citizens on progress. We report on the development of the China results-based Logic Model for Community Health Facilities and Stations (CHS) and a set of relevant PHC indicators intended to measure CHS priorities.

**Methods:**

We adapted the PHC Results Based Logic Model developed in Canada and current work conducted in the community health system in China to create the China CHS Logic Model framework. We used a staged approach by first constructing the framework and indicators and then validating their content through an interactive process involving policy analysis, critical review of relevant literature and multiple stakeholder consultation.

**Results:**

The China CHS Logic Model includes inputs, activities, outputs and outcomes with a total of 287 detailed performance indicators. In these indicators, 31 indicators measure inputs, 64 measure activities, 105 measure outputs, and 87 measure immediate (n = 65), intermediate (n = 15), or final (n = 7) outcomes.

**Conclusion:**

A Logic Model framework can be useful in planning, implementation, analysis and evaluation of PHC at a system and service level. The development and content validation of the China CHS Logic Model and subsequent indicators provides a means for stronger accountability and a clearer sense of overall direction and purpose needed to renew and strengthen the PHC system in China. Moreover, this work will be useful in moving towards developing a PHC information system and performance measurement across districts in urban China, and guiding the pursuit of quality in PHC.

## Background

China, now home to more than 1.3 billion people [1], once had an enviable primary health care (PHC) system which was inexpensive and had a significant impact on population health [2,3]. From 1952 to 1982, China saw rapid improvement in health; life expectancy rose from 35 to 68 years and infant mortality fell from 200 to 34 per 1000 live births[3]. Their approach to health care provided nearly universal health insurance and high accessibility through barefoot doctors to more than 90% of the population[4].

Shortly after 1978, China's universal health insurance collapsed and there was a shift in funding from rural to urban facilities and from PHC to specialized and hospital-based care. This resulted in a proliferation of specialists, a rapid rise in out-of-pocket expenses, excessive use of drugs and high-technology diagnostic tests, decreased access to care and utilization, and a growing health disparity gap between rural peasants and urban city dwellers[5]. However, since 1997 the government has increased health spending, emphasizing PHC renewal through community health facilities in urban China[6], based on evidence that a strong PHC system reduces health inequities across populations[7-10], and may also contribute more to improving population health than specialized health services[10,11].

Although people who need PHC can obtain these services from any health facility (e.g., hospital, clinic, community health facility), the community health sites are especially designed to deliver PHC, providing a basket of comprehensive services designed to address acute and episodic health conditions in order to improve access and continuity of care and increase the overall effectiveness of the health care system[12]. Indeed, the main place of PHC delivery is through the publicly funded community health facilities and smaller, affiliated, community health stations (CHS; we herein refer to community health facilities and stations interchangeably). CHS facilities provide residents health education, family planning and rehabilitation and is a key component of community development[13,14].

The number of CHS facilities almost tripled by 2008, with the proportion of cities offering PHC services reaching 91%, through an estimated 29,127 facilities and 185,050 professionals [15]. The federal government has planned a total of US $124 billion dollars to support the reform of the entire health system [6]. Some of these investments include: US $310 million to renovate 2,400 CHS facilities, US $12.4 million for CHS workforce training, US $34.1 million to build more CHS facilities, and investments which will increase annually with the base of US $2.33 per person in 2009 to pay for basic public health services[16].

Population-based information and reporting systems are needed as policy-makers and managers seek to monitor the performance of PHC, identify areas requiring structural or process modifications, assess the relative impact of different strategies to catalyze renewal and account to citizens on progress[14,17,18]. No national information or reporting system for the community based PHC system currently exists. Although the CHS facilities are required to participate in monitoring and measurement of performance, most of this work is completed at the district and county level and has mainly focused on examining the structure of service delivery (e.g. financing, organization, health human resources, and volume of visits)[19]. This paper reports work conducted in China since 2007 to: 1) develop and modify a Results-Based Logic Model, a performance management and accountability framework for the CHS system and 2) identify CHS priorities in order to develop a useful set of relevant PHC indicators.

### Why use a Logic Model?

A Logic Model depicts the flow of resources and processes required to produce the results desired by the organization or program. Simply put, a Logic Model attempts to visually convey the connection between inputs, activities, outputs, and outcomes[20]. It can offer guidance in the development of an information system [21] by supporting the: a) identification of relevant performance indicators relevant to policy makers and providers and b) development of evaluation or research questions aimed at examining whether performance or health outcomes improves.

A common framework for delivery of PHC affords stakeholders the opportunity to more clearly consider and communicate expected associations and links between goals and objectives, alternative courses of action, and the attainment of results. It defines the areas in which information, evaluation and evidence are needed for policy, administrative and practice communities to plan, monitor, guide and report on PHC renewal[22,23]. Moreover, a PHC Logic Model framework can be useful at the organizational level for planning delivery of services and designing outcomes-based evaluations of programs. Logic Model frameworks can assist policy makers, managers and providers implement targeted quality improvement efforts[22,23].

## Methods

We adapted the Canadian PHC Results Based Logic Model which was developed in response to the lack of a common performance measurement and evaluation framework for understanding the PHC system. More detail about the development and validation of the Canadian PHC Logic Model, using the Treasury Board of Canada results-based management accountability framework, policy analysis, research evidence, and broad consultation with multiple stakeholder groups, can be found elsewhere[22,23]. The PHC Logic Model was chosen to guide this work since it has been used to examine PHC renewal, summarize expected outcomes of PHC, and guide analysis for the simultaneous impact of PHC activities on outputs (e.g., *type *of care such as health promotion and *qualities *of care such as accessibility and comprehensiveness of services) and outcomes throughout Canada and internationally.

### China CHS Logic Model

In order to create the China CHS Logic Model, we used a staged approach. First we constructed the framework and indicators. Then we sought content validation through an intensive interactive process involving policy analysis, critical review of relevant literature and multiple stakeholder consultations. Our definition of PHC in China is based on work by the State Council of China[13], the World Health Organization[24], and internationally recognized academics whose expertise is in the area of PHC. Primary health care is defined as the first contact of care and the delivery of comprehensive, continuous, and convenient episodic and preventive health care services to families. Services are provided by general practice physicians, nurses, public health workers, and other allied health professionals (e.g., pharmacists).

### Policy analysis and literature review

We collected CHS policies related to investment, facilities management, capacity building, register with social health insurance, and so on from the published and grey literature to identify the goals and objectives relevant to CHS service delivery and the role of CHS in China. We then conducted a content analysis of the China national and provincial policy documents, spanning 1997-2008[25-27]. This analysis consisted of a summary of the key points and recurring themes for each topic (e.g. facilities management, capacity building). A review of the literature identified CHS performance frameworks[22,23,28] and relevant indicators used in other countries and international organizations. Major databases such as Medline (PubMed interface) and CINAHL were searched using key words (e.g., performance measurement frameworks and community health, quality improvement frameworks) and MeSH headings. Together, the analysis of policy and literature review provided the foundation from which we developed the initial China CHS Logic Model.

### Stakeholder consultations

A multi-stage iterative feedback and revision process was used for stakeholder consultations. These consultations were undertaken for a period of three months, and the model was continually revised in response to multiple stakeholder consultations. We used our partnership with two health districts, BaoAn and WuHou, to recommend stakeholders who could help refine the logic model. Our partners suggested we conduct focus groups with a range of clinicians, researchers, and managers in each district. Our partners approved a list of potential participants and we then sent letters of invitation to these individuals. Participants were chosen based on their knowledge and expertise about CHS and their region of work in China (east, middle, and west of China).

We conducted a series of four focus groups (n = 24) with CHS providers (n = 6), academics (n = 6) and evaluation specialists and policy makers (n = 12). During the course of the focus groups, our health district partners also suggested that we interview key decision-makers (n = 4). These in-depth interviews included leaders from the Ministry of Health and leaders from two pilot units: BaoAn District Health Bureau and WuHou District Health Bureau. Focus groups were run separately for providers, researchers and evaluation specialists, and policy-makers. Focus groups were conducted by one of the authors (DY). A series of open-ended questions asked about where the logic model categories and whether the connections between the different categories (e.g. immediate, intermediate, and final outcomes) made sense. Examples of questions include: "What does the stabilization of chronic conditions mean to you?" and "Should this be an immediate outcome of services provided by CHS facilities? If so, can you tell me more about your thinking on this?" All data were audio-recorded and summarized. The logic model was refined based on the focus group and interview data.

### Performance indicators

Based on the Canadian Institute for Health Information (CIHI) Pan-Canadian PHC Indicators[29] and our review of existing performance indicators[29-34], we iteratively developed indicators to measure different inputs, activities, outputs, and outcomes in the Logic Model. Development of the indicators was based on input from front-line providers, CHS managers, policy makers, and researchers. Similar to the process followed in refining the Logic Model, another series of four focus groups (n = 34) with providers and CHS managers, academic and evaluation specialists, and policy-makers was conducted. Participants were asked about their roles and responsibilities and what they thought was important in terms of measuring their performance. We used the following criteria for choosing the set of indicators: a) importance and relevance to delivery of PHC in China, b) potential feasibility of obtaining data using a PHC information system, and c) evidence suggesting the delivery of PHC activities and services is linked to outputs or immediate, intermediate, or final outcomes. Similar to the CIHI format[29], we developed detailed specifications for each indicator including: (1) a clear operational definition of the indicator; (2) explicit definitions of the key terms included in the definition; (3) any inclusion or exclusion criteria; and (4) the underlying rationale for each indicator.

## Results

**Figure 1:**
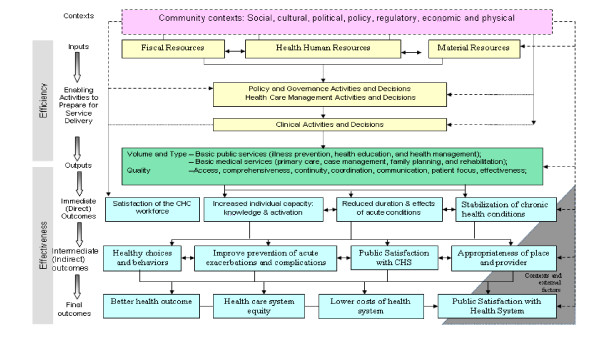
China Community Health Services Logic Model for Performance Measurement of Primary Health Care.

The China results-based CHS Logic Model is a heuristic framework describing relationships between inputs, activities, outputs, and outcomes relevant to CHS (figure 1). While the overarching structure of the framework is similar to that of Canada's PHC Logic Model [34], the inputs, activities, outputs and outcomes of China CHS model are somewhat different. These differences reflect the contextual differences between China and Canada in social, economic, cultural and political arenas and take into consideration China's unique problems confronted by its health system. We describe the China CHS Logic Model here[34].

### Inputs

The foundation of China's PHC system has two parts, resources (or inputs) and activities. Within the social, cultural, political, legislative, and economic and physical context, decision- and policy-makers' attention to community health services are focused on inputs related to fiscal, material, and health human resources. Priorities for improving the renewing of CHS are related to increased financial investment, material resources, and health human resource capacity. Examples of performance measurement input indicators include:

1. Amount of financial investment by the national government for subsidization of services delivered at CHS facilities and capital infrastructure construction in community health; Government leadership that publicly supports the goals of the CHS system and a gradual health system shift to PHC (e.g., percent of sub-districts who have at least one community health centre)

2. Material resources including physical facilities, information technology (e.g., personal health records) and equipment used to support and deliver care. Moreover, the development and widespread adoption of clinical practice guidelines in CHS settings.

3. Number and types of health human resources and their qualifications (e.g., knowledge and discipline-specific competencies, use of interprofessional teams). Examples of types of health human resources needed in delivery of PHC through CHS facilities include general practice physicians, nurses, and nurse practitioners.

### Activities

PHC activities, the second part of the PHC foundation, are processes intended to produce specific outputs and are prepare the PHC system to deliver services. Activities that enable PHC include: policy and governance, health care and clinical management, individual decisions, and community decisions. Priorities for measuring activities related to PHC activities include the need for CHS facilities to accept reimbursement from publicly funded health insurance, increase coordination and collaboration with other facilities to deliver PHC activities, improve the training and continuing education of health human resources working in CHS facilities, and strengthen the capacity of CHS facilities to delivery PHC.

Examples of activity indicators include:

1. Policy and governance: Expansion of the level and coverage of health insurance, enhancing the CHS facilities' ability to accept reimbursement from publicly-funded insurance carriers; Governance structure that strengthens coordination and formal collaboration between CHS facilities and other places (e.g., number of "two-way" referrals by facility where a patient is referred for specialized services and that specialized services (e.g., internal medicine) refer patients to CHS facilities as their place of first contact with the health system;

2. Health care management: Increased use of inter-professional teams, Increasing accessibility of care (e.g., hours and days of operation);

3. Clinical management: Increased training opportunities and continuing education to CHS providers that includes the use of clinical guidelines; Integration between "Western medicine" and Chinese "traditional" medicine

4. Community decisions: Group health promotion activities organized by local community organizations; Support and encouragement by neighborhood committees for communities to use CHS facilities for PHC services.

### Outputs

Services (outputs) are divided into basic public health and medical services with the former more important than the latter. Services can be described in terms of type, volume, distribution, and characteristics. Examples of types of PHC services include: primary, secondary, and tertiary prevention and curative, rehabilitative, palliative, and supportive services. Volume/utilization refers to the amount of different types of services being delivered whereas distribution refers to how the services are allocated to individuals and communities (e.g., who gets how much of what service). The quality of PHC outputs, refers to the degree to which health services for individuals and communities increase the likelihood of desired health outcomes and are consistent with current professional knowledge. Examples of indicators in this area include:

1. Types of services and volume: Percent of CHS facilities that provide public health services such as health education and immunizations; Percent of CHS facilities incorporating rehabilitation, case management (e.g., percent of patient with hypertension who have their care coordinated by a case manager), and use of Chinese traditional medicine;

2. Utilization of CHS for public health and primary medical care (e.g., percent of patients who have a regular doctor located at a CHS facility);

3. Characteristics or qualities of CHS facilities, based on national policy priorities[13], include: Safety, effectiveness, comprehensiveness, continuity (e.g., percent of patients who saw a specialist and have information back to their regular physician within three months), coordination and patient focus.

### Outcomes

PHC outcomes are the results that should occur from the delivery of PHC services. Outcomes can be considered immediate, intermediate, and final. While immediate outcomes should be directly attributable to PHC outputs, intermediate outcomes are those areas in which PHC providers and stakeholders have a lesser degree of control, but for which delivery of quality PHC services are still expected to have some impact. Final outcomes are those areas over which PHC providers and stakeholders have the least amount of control, recognizing that the provision and delivery of health services is only one of the social determinants of health. Examples of priority outcomes include:

1. Immediate: Increased individual capacity, knowledge, and confidence in managing his/her health, reduced duration and effects of acute conditions, stabilized chronic health conditions; Satisfaction of CHS workforce

2. Intermediate: Health related outcomes (e.g., healthy choices and behaviors), patient satisfaction and confidence in CHS facilities, and appropriateness of place and provider (e.g., minimize the use of specialists without a referral from a CHS provider).

3. Final: PHC delivered through China's CHS facilities was designed to attain overall improved population health (e.g., lower premature mortality), equity, and lower overall costs to the health system.

In addition to developing the China CHS Logic Model, we developed a set of performance indicators (n = 287) for China's CHS system which were meant to measure most components of the Logic Model. Each indicator included specifications in order to provide sufficient detail and so that they would be applied consistently across settings. The input indicators (n = 31) were designed to measure China's increasing investment in CHS facilities, health human resources, and funding for PHC. Indicators in the activities component (n = 64) of the model measure factors such as health insurance coverage, education and training, and accessibility. Outputs indicators (n = 105) were designed to measure types and volume of PHC services and the quality of care provided. Given the particularly low utilization of the current CHS in China [35,36], improvements and indicators to measure these improvements are urgently needed. There are a total of 87 indicators that measure immediate (n = 65), intermediate (n = 15), and final (n = 7) outcomes. Although many input indicators are already in use, more regularized use of activity, output, and outcome indicators is needed. While the set of performance indicators may appear daunting, these indicators can be used as a whole or to form subsets of indicators to address different investments or priorities. Depending on the priorities for service delivery via CHS facilities in any given year, a subset of indicators will be selected. Importantly, 24 indicators makeup the core set and used for all routine monitoring of primary care delivered through the CHS. These list indicators can be also used to inform and prioritize the enhancement of the data collection infrastructure over time. Table 1 shows the list of core indicators designed to measure PHC performance and monitor investments in the CHS system.

**Table 1:** Examples of Core CHS Performance indicators

**Category (n)**		**Examples of Core Indicators**	**Source of data**
Inputs (31)	Health Human Resources	• % of qualified health care providers (physicians, nurses, nurse practitioners) in CHS	Health authority records
	Material Resources	• % of sub-districts who have at least one community health center	Health authority records
	Fiscal Resources	• Amount of financial investment for capital infrastructure	Health authority records
Activities (64)	Policy and governance level	• The percentage of CHS facilities that can be reimbursed through publicly funded health insurance	Health authority records
	Health care management level	• % of PHC providers who completed a two-way referral of patients-a patient is referred for more specialized services or services unavailable through the CHS and that more specialized services (e.g., internal medicine) refer patients to CHS facilities as their place of first contact with the health system	Health authority records
	Clinical level	• % of CHS facilities who can offer Chinese traditional medicine	Health authority records
Outputs (105)	Type	• % of PHC organizations who currently provide the following public health services (health education, illness prevention, etc	CHS facility
	Volume	• % of patients with hypertension who have health care coordinated by a case manager	CHS facility
	Quality	• % of patients who have a regular doctor • % of patients who were referred to other doctors and have information back. • % of patients who report that they were given enough time to discuss their feeling, fears and concerns • % of patients who rated the quality of CHS good or excellent	Patient survey
Immediate outcomes (65)	Increased individual capacity	• % of residents who have increased knowledge, skills, and confidence to manager their health	Patient survey
	Reduced risk of ill-health and duration and effects of acute conditions	• Incidence rate of 0-3 year old children with low weight	CHS facility
	Stabilization of Chronic Conditions	• Control rate of patients with chronic diseases (such as hypertension)	CHS facility
	Maintain or improve satisfaction of health care workforce	• CHS provider satisfaction with CHS sector	Provider survey
Intermediate outcomes (15)	Healthy Choices and Behaviors	• % of population who currently engage in regular physical activity	Patient survey
	Improve prevention of complications and acute exacerbations	• Hospitalization rate of patients with chronic diseases	Patient survey
	Public acceptability of CHS	• Patients' satisfaction with CHS	Patient survey
	Appropriateness of place and provider	• % of patients who first see a CHS physician	Patient survey
Final outcomes (7)	Better health outcome	• Decreased premature mortality	National reports (government)
	Health care system equity	• Distribution of health outcome among different populations	National reports (government)
	Lower costs of health system	• Health expenditure per capita in international dollars	National reports (government)
	Public satisfaction with health system	• Residents' satisfaction with health system	Patient survey

The China CHS Logic Model depicts the relationships between inputs, activities, outputs, and outcomes. It also provides a heuristic for PHC decision- and policy-makers to consider the sometimes competing goals of efficiency and effectiveness. Efficiency in the CHS system is a function of the inputs, activities, and outputs[34]. Efficiency is the extent to which an organization, policy, program or initiative is producing its planned outputs in relation to expenditure on resources. Effectiveness is the extent to which the CHS sector delivers its intended outcome or results in a desired process, in response to need[14].

## Discussion

Given that a strong PHC system has a positive impact on population health and reduces the social-economic gradient in health [10,11,37-39] provincial and national governments in China are determined to rebuild a more equitable PHC system to address widespread dissatisfaction and inability to access care due to the shift to a market-oriented health system. Moving PHC delivery out of hospitals into the CHS system and the development of the Logic Model and indicators provides a means for stronger accountability and a clearer sense of overall direction and purpose needed to renew and strengthen the PHC system in China. Although a network of facilities and a general policy framework for CHS has been constructed in the last 10 years, China has lacked a systematic, standardized performance evaluation and management information system. Moreover, no guidance of a consensus-based accountability framework for the CHS system has been used nor did the performance measurement indicators comprehensively cover the quality of care (activities) or immediate and intermediate outcomes. Therefore, these earlier type of evaluations are not as useful in guiding future investments in PHC renewal or in development of China's PHC information system[19].

A Logic Model can be useful in planning, implementation, analysis and evaluation of PHC at a system and service level[23]. This framework was used in two districts in China to generate useful practice information about the relationships between inputs, activities, outputs, and outcomes[35,36]. For example, in one district, we found the incidence of measles was higher than in the past 2 years because immunization to immigrant children was lagging. Using the China CHS Logic Model as a heuristic framework, we found there was a lack of health human resources to administer the immunizations and that there was inadequate structural resources (e.g. a large enough space) to serve the target population. Based on our discussions with the CHS managers, we recommended increasing qualified staff during peak times (e.g., flu season, administration of childhood vaccines) and increasing facility space for future CHS buildings and renovations. Another example is that overall, the coordination between CHS facilities and other services (e.g., specialists, acute care) remains poor. We found there is virtually no communication between CHS facilities and other places where patients go for their care. We are working with CHS managers and specialists to find solutions regarding communication between different sites of care and working with the Chinese government to strengthen the formal structure of care coordination so that the CHS becomes the primary place of care. Importantly, the local Health Bureau of these two districts commended the use of the China CHS Logic Model and its indicators.

While CHS managers generally found the implementation of this heuristic framework and indicators useful in beginning to document their work, more research is needed. We have conducted content validation of the Logic Model and PHC indicator content but will need to conduct more research with policy- and decision-makers, researchers, and evaluation specialists to provide further refinements to the performance measurement framework. Further work is currently underway that will serve to further validate this set of indicators.

The China CHS Logic Model and its indicators will also be used, in part, to guide the development of an information system on measuring the quality and performance of the PHC sector. We will examine how existing population-based data sources can be used to monitor the CHS system, identify gaps in the current data landscape that hinder CHS performance measurement and recommend how these gaps might be filled. Currently, the Ministry of Health is designing the standard National Resident Health Archive[40]. Based on this work, the Community Health Association of China (CHAC) proposed the adoption and use of the China CHS Logic Model and its indicators to the Ministry of Health. It is also expected that CHAC will use the Logic Model to guide complex analysis in order to inform the quality and performance of the CHS system.

## Conclusions

In summary, A Logic Model framework can be useful in planning, implementation, analysis and evaluation of PHC at a system and service level. The development and content validation of the China CHS Logic Model and subsequent indicators provides a means for stronger accountability and a clearer sense of overall direction and purpose needed to renew and strengthen the PHC system in China. Developing the logic model framework and relevant performance measurement indicators has required the articulation of inputs, activities, outputs, and outcomes and extant indicators relevant to PHC renewal in China. Although more work is needed in further refinement of the framework, it will be useful in moving towards developing a PHC information system, comparing common indicators across districts in China, and guiding the pursuit of quality in PHC.

## Competing interests

The authors declare that they have no competing interests.

## Authors' contributions

SW, OB, and DY conceived of the study, participated in its design, developed the initial draft of the Logic Model and performance indicators and drafted the manuscript. DY also conducted the data collection for policy analysis, literature review, and stakeholder consultations. WB and LL participated in the policy analysis and interpretation of the stakeholder consultations. BC is the principal investigator for this project. He obtained the funding, participated in its design and coordination of the data collection. All authors were involved in analysis of data. All authors read and approved the final manuscript.

## Pre-publication history

The pre-publication history for this paper can be accessed here:

http://www.biomedcentral.com/1471-2296/11/91/prepub
